# Coccinellids, Aphids, and Pollen in Diversified Vegetable Fields with Transgenic and Isoline Cultivars

**DOI:** 10.1673/031.007.6101

**Published:** 2007-12-06

**Authors:** G.-A. Hoheisel, S. J. Fleischer

**Affiliations:** ^1^ Washington State University, Presser, WA 99350; ^2^ Pennsylvania State University, University Park, PA 16802

**Keywords:** *Anatis quindedmpunctata*, *Hippodamia parenthesis*, *Hippodamia convergens*, *Coccinella septempunctata*, *Coleomegilla maculata*, *Harmonia axyridis*, sweet corn, winter squash, potato

## Abstract

The influence of concurrent introduction of three transgenic vegetable cultivars on seasonal dynamics of coccinellids and their food, aphids and pollen, was examined within diversified farm systems practicing insect pest management in northeastern US agroecosystems. The transgenic cultivars used included sweet corn, potato, and winter squash, expressing Cry1(A)b, Cry3A, and plant viral coat proteins that target Lepidoptera, Coleoptera, and aphid-transmitted viruses, respectively. Transgenic systems reduced insecticides by 25%. Weekly differences in coccinellid density between transgenic and isoline crops were rare and transitory, governed by timing of at-planting or foliar insecticide use patterns; however cumulative frequencies for three of the six coccinellid species differed between transgenic and isoline crops. At a multicrop, farm systems level, seasonal dynamics of the coccinellids and aphids tracked dynamics in the sweet corn, which far surpassed the other crops in abundance of coccinellids and pollen, and harbored consistently higher aphid densities. Although these results warrant further study, the patterns suggest that diversified transgenic vegetable crops under current commercial management demonstrated transitory conservation of coccinellids, and that integration with selective insecticides or other IPM tactics could increase this potential.

## Introduction

Integrated pest management (IPM) practices strive to advance pest control while minimizing costs and environmental impacts. These management techniques encourage conservation of beneficial organisms while decreasing insecticides. The use of genetically modified organisms (GMOs) in an IPM program may facilitate more targeted, precise pest control. In addition, polycultures have reduced pest pressure while increasing beneficial insect predator densities (Root 1973; Bach 1980; Trujillo-Arriaga and Altieri 1990; Tooker and Hanks 2000). In the northeastern US, vegetable farms are often comprised of interdispersed, small fields, thus approaching polycultures on a farmscale. These farms sell fresh product via direct marketing, and plant multiple vegetable crop species, with multiple planting of these crops. Several crops relevant to northeastern agriculture, including sweet corn, squash, and potatoes, have, or have recently had, commercially available genetically modified cultivars, creating the potential of IPM programs that combining diversified cropping systems and GMOs at a farmscale. Concurrent with the introduction of GMOs, the types of insecticides and their use patterns are rapidly changing, such as the use of neonicotinoids applied at-planting instead of multiple foliar applications in both potatoes and cucurbits, and neonicotinoid seed treatment coupled to transgenic cultivars. Although recent studies have considered epigeal communities in these systems (Mullen *et al* 2005; Leslie et al. 2007), little is known about the effects of GMOs on foliage non-target insects within a diversified farm using current insecticidal materials and use patterns.

Ladybugs (Coleoptera: Coccinellidae), are one non-target taxon that are easily found on diversified farms, and have long been used as a biological control agent of numerous aphid species (Hazzard and Ferro 1991; Dreisdat and Flint 1996; Elliot et al. 2002) in a wide range of crops [pecans (LaRock and Ellington 1996); chili peppers (Votava and Bosland 1996); chrysanthemums (Dreistadt and Flint 1996); alfalfa (Elliot et al. 2002); corn (Andow and Risch 1985)]. Although ecological theories suggest that a more complex plant system will promote a greater diversity of prey and more efficient predator populations (Root 1973; Bach 1980), coccinellids do not consistently follow this dictum (Andow and Risch 1985). In monocultures, prey and pollen sources are more abundant and evenly distributed than polycultures, resulting in higher *Coleomegilla maculata* (De Geer) populations (Andow and Risch 1985). Higher coccinellid populations in monocultures suggest that coccinellids are regulated by the food quantity as opposed to quality (Andow and Risch 1985).

For many coccinellid species, aphids are essential for their optimum development and fecundity (Baumgaertner et al. 1981; Kalaskar and Evans 2001; Kalushkov and Hodek 2001), and C. *maculata* develops faster when consuming large numbers of aphids (Chedester 1979). In addition to prey, coccinellids eat pollen for a rich source of protein. In some species, egg predation decreases when sweet corn pollen is available as an alternative food source (Pfannenstiel and Yeargan 2002). Direct toxicological effects on coccinellids from ingestion of the coleopteran-targeting Cry3Bb proteins through field corn pollen has been negligible (Duan et al. 2002; Lundgren and Wiedenmann 2002; Al-Deeb and Wilde 2003). Nonetheless, a diet solely of pollen does not produce the highest rate of development (Riddick and Barbosa 1998), suggesting that a diet of plant and insect material is optimal.

When transgenic cultivars are incorporated into an agroecosystem, pesticide use patterns and prey communities are changed, which might influence the population dynamics of coccinellids through direct or indirect effects. Foliar applications of insecticides, can either increase aphid populations by removal of natural enemies, or reduce aphid populations through direct toxic effects. At a single crop level, previous studies found no changes between coccinellid populations in transgenic and non-transgenic potato (Riddick et al. 1998) and corn (Pilcher et al. 1997). In these studies, an alternative prey or pollen food source was abundant in the transgenic crops.

In this study, the influence of concurrent introduction of three GMO vegetable cultivars, as part of an IPM program, on seasonal dynamics of coccinellids and their food, aphids and pollen, was investigated within a setting relevant to commercial diversified farms in northeastern US agroecosystems. The plots studied included the isoline varieties of sweet corn, potatoes, and winter squash and their transgenic cultivars that protect against lepidopterans, *Leptinotarsa decemlineata* (Say), and aphid-transmitted viruses, respectively. We hypothesized that the host plant will affect insect population dynamics, and that crops with the highest pollen and prey loads should contain the most coccinellids, while the seasonal dynamics and predator-prey relationships will not differ between agroecosystems using the transgenic versus isoline cultivars using IPM.

**Figure 1.**
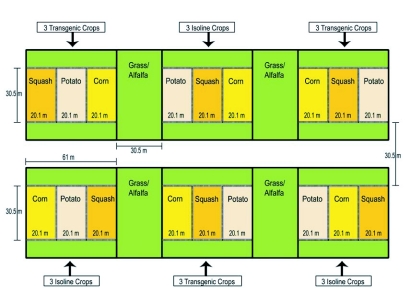
Split-plot experimental design consisting of 3 replicates of two genotypes: transgenic and isoline. Main plots were split among 3 crops consisting of sweet corn, potatoes, and winter squash. Transgenic cultivars targeted Lepidoptera in corn, Coleoptera in potato and aphid-transmitted viruses in squash.

## Materials and Methods

### Experimental design

Three acres of vegetables, consisting of sweet corn, acorn squash, and potato, were grown in a split plot design at the Russel E. Larson Experimental Farm in Rock Springs, Pennsylvania in two main plots, a transgenic and its isoline, were replicated three times. Each main plot was split among the 3 crops: sweet corn, acorn squash, and potatoes (Figure 1). An alfalfa/grass mix formed the borders of all plots, and this was kept mowed. Main plots were 61 m × 30.5 m (0.186 hectares) and subplots were 20.3 m × 30.5 m (0.062 hectares). Cultivars (isoline and transgenic, respectively) consisted of ‘Jackpot’ and ‘BC 0801’ expressing the Cry1(A)b endotoxin in the sweet corn, ‘Taybelle’ and ‘Taybelle PM’ expressing plant viral coat proteins for the acorn squash, and ‘Superior’ and ‘New Leaf Superior’ expressing the Cry 3A endotoxin for the potatoes. The corn and potatoes were planted with a 0.30 m seed spacing totaling 100 plants/row or 2600 plants/corn subplot (0.76 m row spacing) and 1900 plants/potato subplot (0.91 m row spacing). The squash was planted in plastic mulch for weed control and spaced at 1.22 m for a total of 25 plants/row or 275 plants/subplot (1.83 m row spacing). The total crop area for corn, potato, and squash was 19.8 m^2^, 17.4 m^2^ and 20.1 m^2^, respectively. This experiment was conducted in 2001. The potato, corn and squash were planted on May 8, May 29, and June 6, respectively.

Both transgenic and isoline main plots were managed using IPM practices: insecticides were applied when the average pest population among all replicates of a main-plot treatment for that crop reached the predetermined thresholds described by Foster and Flood (1995). Pests were monitored twice a week by scouting 10 plants/subplot in all crops, using sweep nets in potatoes, and wire cone pheromone traps baited with Zealure (Hercon Environmental, www.herconenviron.com) to monitor corn earworm pests. All insecticides were applied following label recommendations, using the midpoint of the labeled rate range. At planting, imidacloprid (Admire 2F) was applied to the isoline potatoes to control Colorado potato beetle, and diazinon (Germinate PL) was applied to both genotypes of corn to control seed corn maggot (Table 1) to replicate current local farming practices. Fungicide and herbicide treatments did not vary among treatments, and are detailed in Hoheisel (2002).

**Table 1.**
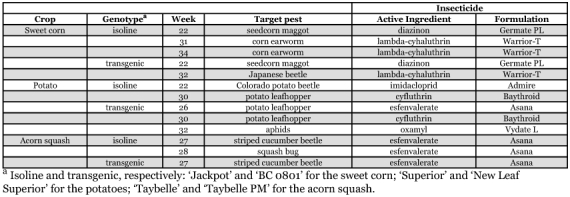
Insecticide use pattern in diversified transgenic and isoline vegetable crops. Applications were applied when mean density of target pest among all subplots exceeded pre-determined thresholds.

### Coccinellid, Aphid, and Pollen Densities

Adult coccinellid and aphid densities were determined with whole plant searches along the stem and on both sides of all leaves. Coccinellids were identified to species level, and vouchers placed in the Frost Entomological Museum, Pennsylvania State University. Aphid taxa were identified to family. Scouting for coccinellids was done twice a week. From 26 June to 12 July, 1/8 of the field was scouted in all crops. From 16 July to 16 August, the squash and potato subplots were examined entirely due to low abundance while the corn subplots remained at 1/8 of the field. Densities were standardized to numbers/m^2^/week.

Aphid abundance was determined twice a week for the first three weeks and once a week for the last five weeks, using 10 plants/subplot. The number of aphid colonies was counted within five predetermined size classes; O, 1–25, 26–50, 50–100, >100 individuals. The number of aphids/m^2^/week was calculated by multiplying the number of colonies within a category by the mean of the category. For colonies >100, 125 was used for the mean.

Pollen collection techniques varied among crops. Sweet corn, which is wind pollinated so that much of the pollen available to coccinellids on the leaf surfaces, was collected by pressing a microscope slide bearing double-stick tape onto a leaf. Potato pollen is only released upon stimulation by a vibration similar to the frequency of a bee. Thus, the pollen was extracted by vibrating a tuning fork against the flower and the pollen caught on a microscope slide with double-stick tape. Squash pollen primarily remains on the anther until a pollinator enters the flower. Therefore, squash pollen was collected by rolling a slide with double-stick tape around the anthers. The number of pollen grains within 1 cm^2^ of each slide was counted for 5 plants/subplot/week (n=15 for each main-plot genotype treatment). For potato and squash, the pollen/m^2^ was extrapolated from the number of pollen grains/flower, flowers/plant and plants/m^2^. Pollen/m^2^ in corn was calculated from the quantity of pollen collected, cm^2^/leaf, leaves/plant and plants/m^2^.

Sweet corn leaf area (cm^2^/leaf) was calculated from the average of 20 leaves within each subplot; (n = 60 for each genotype). The corn leaves were traced on paper and the drawings were cut out and weighed. To convert weight to area, a mean from 50, 10 cm^2^ samples of paper, were weighed (ū_weight_ = 0.77 ±0.013). The corn leaves/plant were calculated by averaging the number of leaves on 5 plants per subplot (n = 15 per genotype). The anther/flower in squash was a constant of one. In potato and squash, the flowers/plant were found by averaging the number of flowers, using male flowers only in squash, on 20 plants per subplot (n = 60 for each genotype).

### Analyses

The seasonal dynamics of coccinellids, aphids, and most pollen data were examined on both a multicrop (main plot, or genotype) level, and a per crop level using a generalized linear model with a Poisson distribution and a natural log transformation. The squash pollen followed a negative binomial distribution (SAS 1999). To adjust for underdispersion, the variance was fitted with a deviance factor so that the empirical variance was used to calculate p-values. Significance was determined by the differences in least square means for each genotype by week interaction.

**Table 2.**
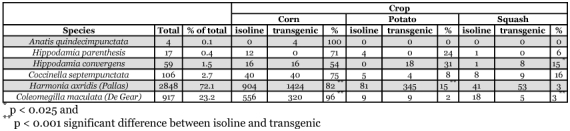
Frequency distribution of coccinellids among transgenic and isoline genotypes of three vegetable crops.

The frequency distribution of the total coccinellids per crop and genotype was analyzed using a G-test. The G-statistic was adjusted using William's correction for two-cell cases (Sokal and Rohlf 1995).

## Results

### Insecticide frequency

At a multicrop farm-scale level, 8 applications were applied in the isoline, and 6 in the transgenic cultivars (Table 1). There were more insecticides applied to the isoline genotypes of corn (3 versus 2), and squash (2 versus 1). Inversely, transgenic potatoes received more insecticides than isoline (3 versus 2).

### Coccinellid community composition and distribution

**Figure 2.**
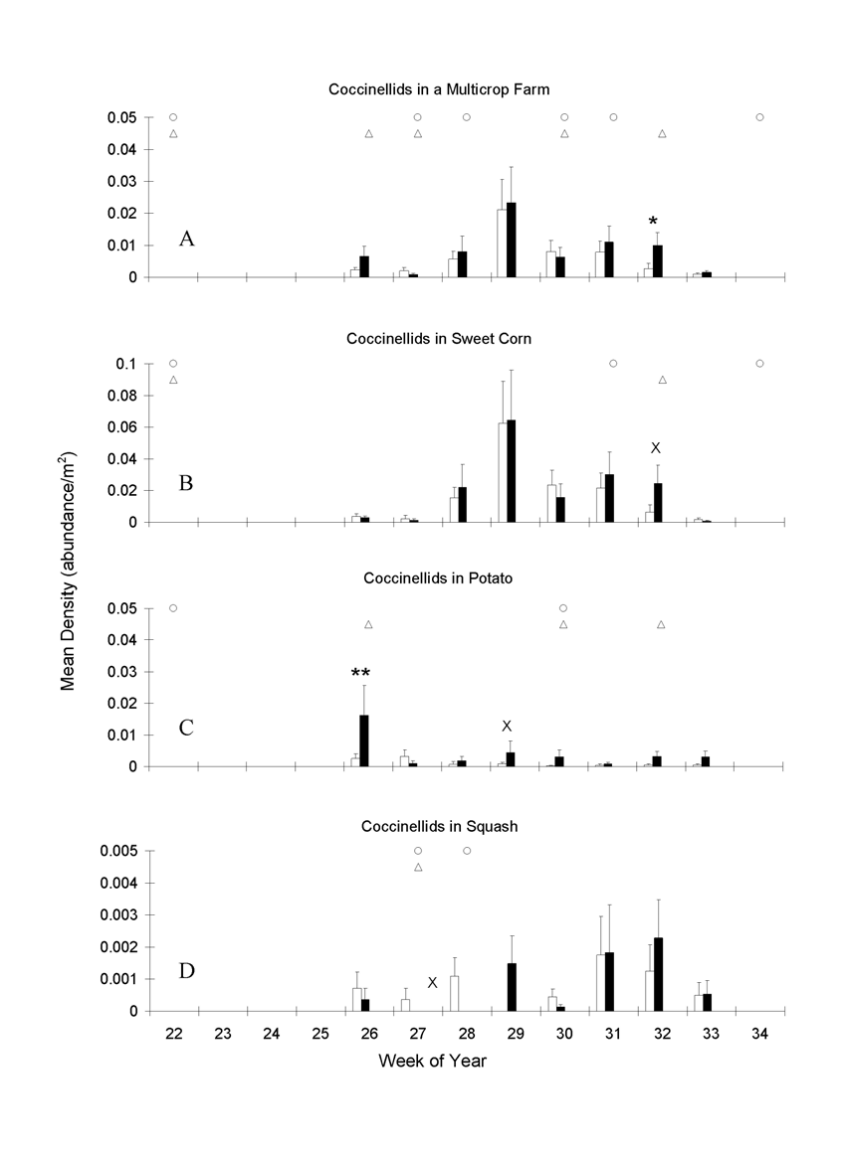
Seasonal dynamics of Coccinellidae at a multicrop (main plot) level, and within each of three crops. Week 26 is July 26, 2001, white bars = isoline, black bars = transgenic, x = p<0.1, * = p<0.05, ** = p<0.01. Timing of at-planting and foliar insecticide applications (see Table 1) noted by circles in isoline and triangles in transgenic cultivars. Note differences in scale among crops.

The coccinellid community was comprised of six species (Table 2). Two species, (*Harmonia axyridis* (Pallas) and *C. maculata*) were dominant, and represented 72% and 23% of the community, respectively. *Coccinella septempunctata* L. represented an additional 2.7%, and the three other species, *Anatis quindecimpunctata* Olivier, *Hippodamia parenthesis* Say, and *Hippodamia convergens*, Guerin-Meneville were rare (<2% of total community composition). Total season-long densities were consistently higher in sweet corn for all taxa (Table 2, Figure 2).

Within a given crop, all species displayed varying distributions among the genotypes when examined across the entire season (Table 2). There were significantly more *H. axyridis* in the transgenic sweet corn and potato than in the respective isolines (G = 117.11, p < 0.001, d.f. = 1, corn; G = 175.92, p < 0.001, d.f. = 1, potato). In contrast, *C. maculata* was more abundant in the isoline sweet corn and squash (G = 64.34, P < 0.001, d.f. = 1, corn; G = 7.63, p < 0.01, d.f. = 1, squash). *H. convergens* occurred more frequently in transgenic than isoline squash (G = 5.87, p < 0.025, d.f. = 1), but their overall abundance was low in this crop (n = 9). *A. quindecimpunctata* was only found in transgenic corn, while *H. parenthesis* was found only in the isoline crops. *H. convergens* was more evenly distributed among the crops than any other species (54% corn, 31% potato, 15% squash), yet there were no individuals in the isoline potato and only one in the isoline squash.

### Seasonal dynamics

**Figure 3.**
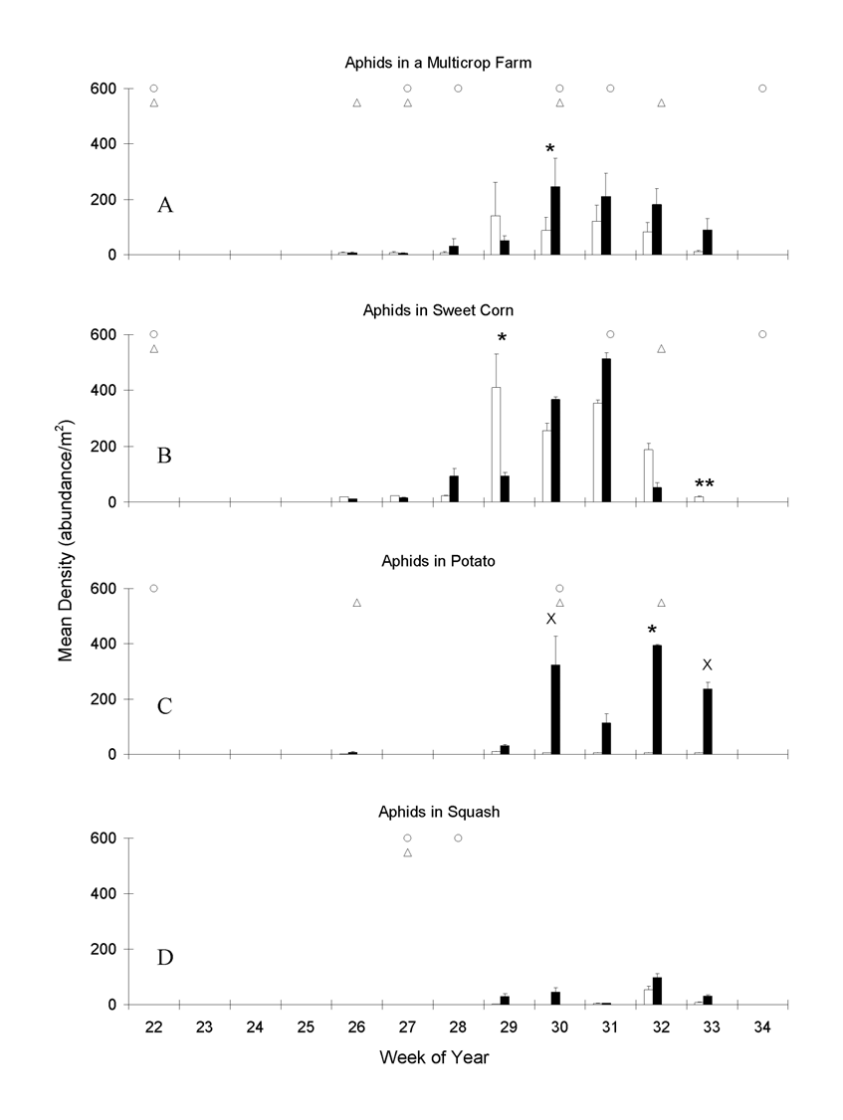
Seasonal dynamics of Aphididae at a multicrop (main plot) level, and within each of three crops. Week 26 is July 26, 2001, white bars = isoline, black bars = transgenic, x = p<0.01, * = p<0.05, ** = p<0.01. Timing of at-planting and foliar insecticide applications (see Table 1) noted by circles in isoline and triangles in transgenic cultivars. Note differences in scale among crops.

The aphid populations in transgenic and isoline multicrop farms showed an increase between weeks 29 and 32, after which they began to decrease (Figure 3a). Only week 30 showed a significantly higher abundance of aphids in the transgenic fields (χ^2^ = 4.09, p = 0.0431, d.f. = 1), which corresponded to high densities in transgenic potato and both corn genotypes. Aphid populations were clustered between weeks 29 – 33 in all crops, and while densities were again highest in sweet corn, they were more evenly distributed among at least one genotype of all crops than were coccinellids.

Aphid populations in the isoline sweet corn peaked earlier than those in transgenic fields, resulting in a significant difference between genotypes in week 29 (Figure 3b). The populations began to decline during weeks 32 and 33, which were preceded by insecticide sprays. Only two of the eight weeks showed a significant difference in the genotypes (χ^2^ = 5.50, p = 0.0190, d.f. = 1, week 29; χ^2^ = 213.96, p < 0.0001, d.f. = 1, week 33) and in both cases, aphid densities were higher in the isoline cultivar.

Aphid populations were extremely low in the isoline potatoes, but peaked in the transgenic fields during the last four weeks of the season in which densities were comparable to those in sweet corn (Figure 3c). This resulted in one week with significantly higher populations in transgenic fields (χ^2^ = 4.21, p = 0.0402, d.f. = 1, week 32) and two weeks that were nearly significant (χ^2^ = 3.42, p = 0.0644, d.f. = 1, week 30; χ^2^ = 3.19, p = 0.0740, d.f. = 1, week 33). Not unexpectedly, aphid populations decreased in the weeks following an insecticide treatment.

Aphids were always at low densities in the squash (Figure 3d), yet small peaks coincided with peaks seen in the corn and potato during weeks 29 to 32. No significant differences were seen between the squash genotypes in any of the weeks.

**Figure 4.**
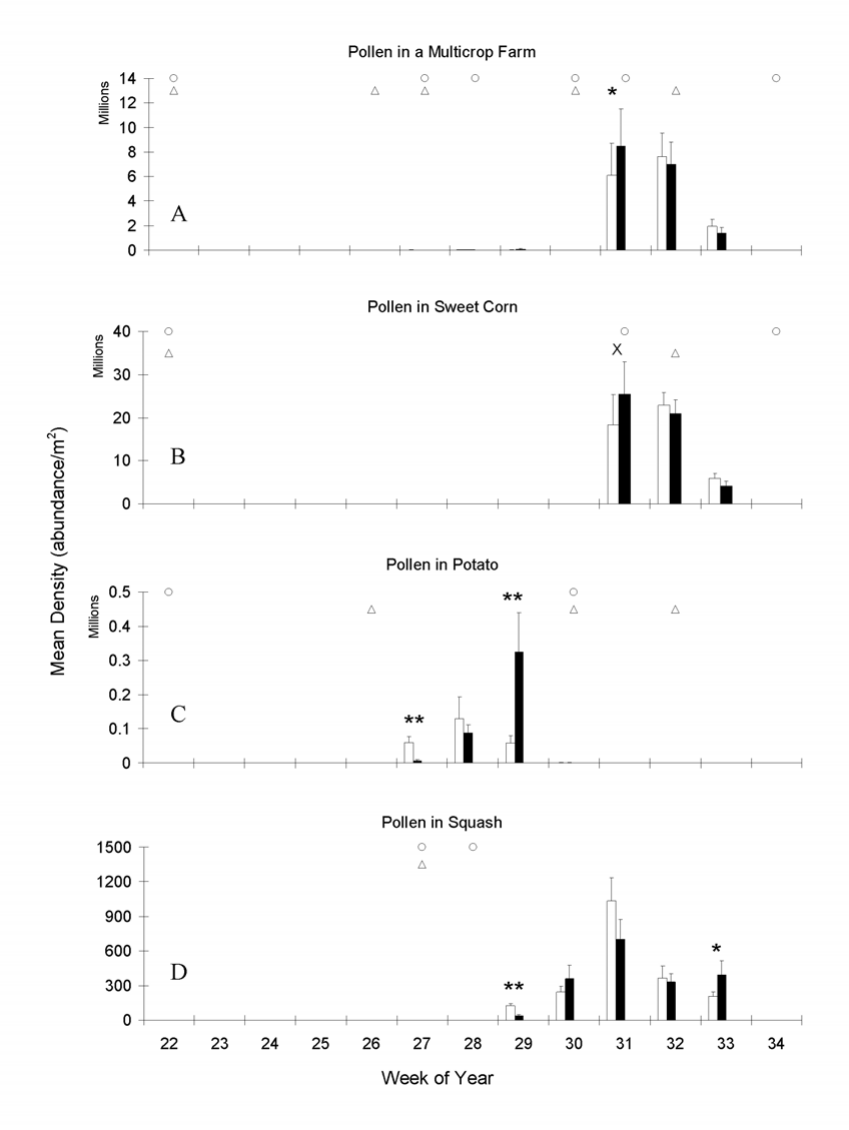
Seasonal dynamics of pollen at a multicrop (main plot) level, and within each of three crops. Week 26 is July 26,2001, white bars = isoline, black bars = transgenic, x = p<0.1, * = p<0.005, ** = p<0.01. Timing of at-planting and foliar insecticide applications (see Table 1) noted by circles in isoline and triangles in transgenic cultivars. Note differences in scale among crops.

Small levels of pollen were available in the multicrop farm beginning at week 27, but high levels were not seen until week 31 (Figure 4a). The dynamics of pollen in a multicrop farm and the nearly significant difference in week 31 (χ^2^ = 2.81, p = 0.0939, d.f. = 1) were almost identical to the seasonal dynamics of pollen in sweet corn (Figure 4b). Sweet corn had approximately 10 times more pollen grains per m^2^ than potato (Figure 4c) and 3000 times more than squash (Figure 4d). Additionally, the sweet corn density is a conservative estimate, since the amount of pollen available to coccinellids on the leaves was measured, not the amount of pollen produced by the anthers.

For each crop, genotype did not differ in the total pollen production; however, the timing of pollen production did differ between genotypes (Figure 4b, c, d). There was a difference between genotypes in the initial pollen production for potato and squash (χ^2^ = 7.56, p = 0.0060, d.f. = 1, potato weeks 27; χ^2^ = 19.64, p < 0.0001, d.f. = 1; squash week 29), while sweet corn approached levels of significant differences (χ^2^ = 3.31, p = 0.0689, d.f. = 1, corn week 31). Sweet corn was the only crop with more initial pollen in the transgenic genotype. The majority of the transgenic corn was silking (73%) while the isoline was in tassel (67%). During the first week of flower production isoline potato produced greater than 8 times more flowers (isoline=441, transgenic=53) and subsequently more pollen than transgenic potatoes. However, the transgenic potato continued to produce more pollen later in the season (χ^2^ = 37.56, p < 0.0001, d.f. = 1, week 29). Isoline squash fields began with higher pollen densities, but on the last week of sampling, transgenic fields had significantly higher pollen densities (χ^2^ = 6.18, p = 0.0129, d.f. = 1, week 33). Similarly, more flowers were seen in the isoline squash initially (isoline=8 transgenic=0, week 28), while the transgenic squash continued flowering later in the season (isoline=73 transgenic=127, week 33).

Seasonal dynamics for each coccinellid species were similar to all species combined, and the combined results are reported here. Coccinellids at a multicrop level showed unimodal curves in both the transgenic and isoline fields (Figure 2a). Six of the eight weeks showed no significant difference between the two genotypes in coccinellid abundance. Week 26 showed a nearly significant difference in coccinellid populations (χ^2^ = 2.98, p = 0.0843, d.f. = 1), while populations differed greatly at the end of the season (χ^2^ = 5.87, p = 0.0154, d.f. = 1, week 32). Differences among genotypes in these two weeks can be associated with peak populations in individual crops.

Within each crop, coccinellids had different seasonal dynamics (Figure 2b-d). Coccinellids in potato peaked first during week 26. Coccinellids in sweet corn and squash reached peaks in subsequent weeks (week 29 corn, week 31 and 32 squash). When comparing peak densities, there were approximately 3 times fewer coccinellids in transgenic potatoes than sweet corn, and 20 times fewer in isoline potato.

Coccinellids in transgenic and isoline sweet corn increased rapidly to a peak at 29 weeks (Figure 2b) which coincided with the majority of the crops being in pre-tassel (week 28). The populations declined slowly over the next four weeks, and this decline started prior to any foliar sprays. Only week 32 showed any slight significant difference (χ^2^ = 2.97, P = 0.0848, d.f. = 1) in which coccinellids were more abundant in transgenic fields. In the preceding week, the isoline sweet corn had been sprayed for corn earworm (Table 1).

Coccinellids in transgenic potatoes were readily apparent at their peak (week 26), but declined rapidly after an insecticide treatment for potato leafhopper was applied. In contrast, populations in isoline potato, which received a neonicotinoid application at planting, remained at low densities (Figure 2c). Population densities in the transgenic cultivar were significantly higher at the start of the season (χ^2^ = 12.65, P = 0.0004, d.f. = 1, week 26) and only approaching significance in one other week (χ^2^ = 3.34, P = 0.0678, d.f. = 1, week 29). Densities were similar between genotypes for all other weeks.

Squash had the fewest coccinellids of all the crops (Figure 2d). Coccinellids increased later in the season during weeks 31 and 32 in which the majority of the crops were flowering and fruiting. There was no significant difference in coccinellid densities among genotypes during any week.

## Discussion

The overall number of pest outbreaks and insecticide treatments between genotypes were similar within a crop and only differed by one additional spray in each crop. Yet on a multicrop scale, insecticide applications were reduced by 25% in the transgenic cultivars compared to isoline. In sweet corn, the CryI(A)b protein protects against European corn borer and, to a lesser extent, corn earworm (Burkness et al. 2001), However, the number of pests was low and only 2 insecticide applications to the isoline were necessary, No foliar applications were applied to transgenic sweet corn for lepidopterans, but one spray was needed to control the Japanese beetle, While a reduction in application frequency was observed, further work with more years and locations is warranted to generalize about the influence of multiple transgenic crops on choice and frequency of soil and foliar insecticides at a farm scale.

Seasonal dynamics of the organisms we monitored at a multicrop scale resembled the dynamics of these organisms in the most dominant crop, Sweet corn far surpassed the other crops in abundance of coccinellids and pollen, and also harbored consistently higher aphid densities, This is consistent with Groden et al, 1990 who reported higher *C. maculata* densities in pollinating sweet corn relative to other vegetable crops, The fact that coccinellid populations were four times higher in sweet corn than other crops coincides with Andow and Risch's (1985) observations that ladybugs are often more abundant in areas with high densities of corn pollen and aphids, and food quantity, rather than quality, is a more dominant force driving their biology, This idea is further emphasized by the low densities of coccinellids in transgenic potato, which had comparable levels of aphids to those in corn, yet its pollen is not a typical food source for ladybugs, Since we did not directly measure coccinellid movement, reproduction or survivorship, it is difficult to decipher if sweet corn has the potential to increase or decrease coccinellid populations in neighboring crops, Nonetheless, high densities in corn imply that efforts at conserving coccinellids as biological control agents in multicrop vegetable farms need to emphasize conservation in sweet corn, The two most abundant species, *H. axyridis* and *C. maculata*, peaked in corn during pretassel to very early tassel, prior to pollination, possibly responding to rising aphid densities during that time, If possible, withholding insecticide applications or use of selective materials during this time could enhance conservation of coccinellids at a farmscale.

Significant differences in coccinellid densities on any given week between genotypes were rare and transitory, and were primarily governed by timing of either foliar or at-planting insecticide applications, The greater early season abundance in transgenic potato is likely a result of both slightly higher aphid populations obtained without an imidacloprid seed treatment and low densities of food sources in other crops, Similarly, the lower abundance in isoline sweet corn during week 32 appears more related to an insecticide spray the previous week than pollen or aphid densities, which were not significantly different during week 32, In all the crops, the majority of the weeks show no difference in coccinellid density between the two genotypes, When differences occurred, they showed higher densities in the transgenic crops and cumulatively there were more coccinellids in the transgenic cropping system, Together, these patterns suggest that diversified GMO crops under current commercial management demonstrated transitory conservation of coccinellids, and that integration with selective insecticides or other IPM tactics could increase this potential, Yet, the lack of differences between genotypes suggests that standard IPM practices are effective in maintaining coccinellid populations and that timing of insecticide applications, rather than genotype, is more influential on the population.

Relative density of the coccinellids varied greatly among species, and, surprisingly, the seasonal total frequency of some species was higher in a particular genotype, There were significantly more *H. axyridis*, the most abundant coccinellid, in the transgenic corn and potato, In contrast, the second most abundant species, *C. maculata*, occurred more frequently in the isoline corn and squash, These observations cannot directly explain the differences in abundance as numerous factors can drive the ecology of coccinellids, The species-level taxonomy of the aphids was not determined, and coccinellid species may respond differently to different aphid species, as well as alternative prey such as lepidopteran and coleopteran eggs not recorded in this study, The two most abundant species *H. axyridis* and *C. maculata* have shown differences in their prevelance to consume pollen versus aphids (Lundgren et al, 2004), which could affect distributions among genotypes and host plants, In addition, phenological and tritrophic interactions among the plants, aphids and coccinellids that differ among the genotypes could result in preferential crop partitioning among species of ladybugs.

In this study, the differing pollen levels within a single crop did not correspond with significant variation in coccinellid populations, possibly due to saturation of food sources. Nonetheless, the data suggest that genotype has an affect on the phenology of the plant. There were no clear trends among crops as to which genotype produced more initial flowers and pollen, suggesting the effects are crop dependant. Further studies are needed to determine the extent of this occurrence and how it changes the dynamics of pollenivorous insects. This study was conducted for a single year. We acknowledge that insect populations vary annually, and the conclusions derived from comparisons among densities of predators and prey and resulting insecticide input need further evaluation. Nonetheless, we suggest that the relative abundance of coccinellids among these crops will probably remain similar among years. It is our hope that this study will spawn further ecological work, not only on the coccinellid community, but also on their conservation and movement as related to IPM.

## Abbreviations

IPM -: integrated pest management
GMO -: genetically modified organisms
